# Comparing the in vitro efficacy of chlorhexidine and povidone-iodine in the prevention of post-surgical endophthalmitis

**DOI:** 10.1186/s12348-024-00404-2

**Published:** 2024-05-23

**Authors:** Celso Soares Pereira Batista, Irene Loscos-Giménez, María Gámez, Raul Altaba, Daniela de Miniac, Neus Martí, Francisca Bassaganyas, Elena Juanes, Alba Rivera, Ferran Navarro

**Affiliations:** 1 Microbiology department, Hospital de la Santa Creu i Sant Pau, C/ Sant Quintí, 89. Planta B-2, Barcelona, 08041 Spain; 2 Department of Genetics and Microbiology, Autonomous University of Barcelona, Barcelona, Spain; 3 Ophthalmology department, Hospital de la Santa Creu i Sant Pau, Barcelona, Spain; 4 Pharmacy department, Hospital de la Santa Creu i Sant Pau, Barcelona, Spain; 5 Sant Pau Biomedical Research Institute (IIB Sant Pau), Barcelona, Spain

**Keywords:** Chlorhexidine, Endophthalmitis, Intravitreal injections, Povidone-iodine

## Abstract

**Background:**

Intravitreal injections are a common ophthalmologic procedure. While infections following these injections are rare, they can lead to endophthalmitis, with potentially serious consequences. Various methods have been proposed to prevent endophthalmitis, including the use of antisepsis and antibiotics in patient preparation.

**Purpose:**

To evaluate the antiseptic efficacy of aqueous chlorhexidine (CHX) and povidone-iodine (PI) when used alone and in combination with lidocaine gel (LG) in vitro.

**Methods:**

Two independent experimental trials were conducted. The first trial determined the minimum inhibitory concentrations (MICs) and the minimum bactericidal concentrations (MBCs) of CHX and PI against six bacterial strains. The second trial evaluated the bactericidal efficacy of the antiseptic agents (CHX 0.1% and PI 5%) and their combination with LG against the same bacterial strains.

**Results:**

CHX was more effective than PI in reducing the number of colonies forming units (cfus) of the tested bacteria. The order in which the antiseptic and LG were administered affected their effectiveness, with CHX administered before LG resulting in greater reduction of bacterial growth.

**Conclusions:**

CHX 0.1% is more effective than PI 5% as an antiseptic agent. Application of CHX and PI prior to the use of lidocaine gel results in a more effective reduction of microorganisms.

**Supplementary Information:**

The online version contains supplementary material available at 10.1186/s12348-024-00404-2.

## Introduction

The intravitreal injection (IVI) is one of the most commonly performed procedures in ophthalmology [1, 2]. While infections following these injections are rare, 1 in 1000–5000 cases leads to endophthalmitis [3, 4], which can have serious consequences. The exact mechanism by which postoperative intraocular endophthalmitis (PIE) develops remains unclear, although the patient’s ocular or periocular microbiota is thought to be a significant source of infection [5]. The causative organisms can gain entry into the sterile field through various pathways, including eyelashes or eyelids, respiratory droplets, the injection needle, or contaminated medication [6].

Studies have consistently demonstrated that *Staphylococcus* spp. present on the patient’s ocular surface and conjunctiva are the most frequently identified causes of PIE [7]. However, a meta-analysis of cases separated in time and location [8] revealed a statistically significantly higher proportion of PIE caused by *Streptococcus* spp. in the context of IVI compared to surgical procedures, suggesting that the oral microbiota plays a role in the pathogenesis of PIE [9, 10]. Various methods have been proposed to prevent endophthalmitis, including the use of antiseptics, such as povidone-iodine (PI) or chlorhexidine (CHX), and antibiotics for patient preparation, although antibiotics are currently neglected [11].

PI is an iodophor, a chemical complex composed of a water-soluble povidone polymer and iodine [12], which has been used as a topical antiseptic for several decades. Upon dissolution in water, iodine is released and can penetrate microorganisms, causing cell death by oxidation of cellular proteins, nucleotides, and fatty acids. Chlorhexidine (CHX) is a cationic biguanide, which has been employed as a topical antiseptic since 1954 and is also commonly used as a broad-spectrum antimicrobial agent. Its mode of action involves binding to and disrupting the bacterial cell wall, damaging the semipermeable cytoplasmic membrane and cytoplasm [13]. At lower concentrations, CHX exhibits bacteriostatic properties by displacing cations and destabilizing the cell wall. At higher concentrations, it causes a complete loss of cellular structural integrity, with bactericidal effects. Both PI and CHX are described as active against a wide range of microorganisms, including gram-positive and gram-negative bacteria, fungi, and viruses [12, 13].

It has been demonstrated that the use of PI 5% as the antiseptic agent in cataract surgery is associated with a lower incidence of postoperative endophthalmitis compared to silver protein solution [14]. Accordingly, PI 5% is now part of the standard of care for cataract surgery, and it has been described as suitable for intravitreal therapy [15]. However, there are limited data on the optimal method of PI application in this context.

The use of CHX is discouraged in ophthalmic procedures due to its potential toxicity to the corneal endothelium [1]. However, this toxicity is restricted to alcohol-based CHX and animal models have demonstrated the safety of aqueous CHX on the corneal epithelium as well as its bactericidal efficacy [16]. CHX can be considered a good alternative to PI for antisepsis in patients undergoing IVIs and is reported to cause less discomfort, as measured by a pain perception scale (3/10 vs. 8/10 with povidone-iodine) [17]. The efficacy of CHX is similar to that of PI in terms of ocular bacterial count after antisepsis [18]. In a multicenter retrospective case series, the endophthalmitis rate when using aqueous CHX was 0.0074%, which is comparable to that of PI [18]. Both antiseptic agents have drawbacks. The use of CHX has been associated with resistance in methicillin-resistant *Staphylococcus aureus* and fungi [19], whereas PI can cause significant postoperative ocular surface irritation [17]. In addition to the non-demonstration of superiority of one antiseptic over the other, there is no standardized anesthetic protocol available for IVIs [20].

In this study, the antiseptic efficacy of aqueous CHX and PI, used alone and in combination with lidocaine gel (LG), was evaluated in vitro.

## Methods

Two independent experimental trials were conducted. The first trial determined the minimum inhibitory concentrations (MICs) and minimum bactericidal concentrations (MBCs) of the two chemical disinfectants, aqueous CHX and PI, against six control strains. In the second trial, bacterial killing assays were performed with CHX, PI, and LG, applied separately and in combination.

The assays were carried out using the control strains *Staphylococcus epidermidis* (ATCC 12,228), *Staphylococcus aureus* (ATCC 29,213), *Enterococcus faecalis* (ATCC 51,299), *Escherichia coli* (ATCC 25,922), *Pseudomonas aeruginosa* (ATCC 27,853) and *Streptococcus agalactiae* (ATCC 13,813). All them were subcultured in blood agar plates (Biomerieux, Marcy-l’Etiole, France) incubated at 37ºC with 5% CO_2_ 24 h before use. The antiseptic solutions were of CHX 0.1% (BOHMCLORH, Madrid, Spain), PI 5% (CURADONA, Lainco, Barcelona, Spain) and LG (Ophtesic 20 mg/g LDD, Puteaux, France).

### Microdilution test

MICs of CHX and PI were determined by the broth microdilution method following EUCAST guidelines in concentrations in the range of 0.0512 − 0.000025% (512–0.025 µg/ml) for CHX, and 2.5-0.0195% (25,000–195 µg/ml) for PI. MBCs were determined according to a standard protocol [21].

For each strain, four to five colonies were selected and transferred into 5 mL of sterile saline solution, and then adjusted to a 0.5 McFarland turbidity standard (about 1.5 × 10^8^ colony forming units per mL). One hundred microliters of this solution was then transferred to 9.9 mL of BLL Mueller-Hinton Broth (BD, Becton Dickinson, US) for all control strains except for *S. agalactiae*, for which Mueller-Hinton Broth with lysed horse blood (BD, Becton Dickinson, US) was used. The tests were conducted in 96-well microtiter plates, with each well containing 50 µl of the antiseptic dilution and 50 µl of the test organism suspension, resulting in a final concentration of about 5 × 10^5^ colony forming units (cfu) per mL. The plates were incubated for 18–24 h at 37 ºC. After incubation, the plates were read for turbidity and MICs were determined. The MBC was determined by transferring 10 µl from each well showing no growth in the blood agar plates. The MBC is defined as the lowest concentration resulting in ≥ 99.9% killing [21].

### Bacterial killing assays

The bacterial killing assay was conducted at two concentrations for each control strain (10^3^ and 10^4^ cfu/ml) and twice for each concentration. For each dilution, 100 µl was inoculated onto blood agar plates. After the plates had been inoculated and allowed to dry, they were divided into ten groups: no treatment (1), treatment with LG (2), treatment with CHX (3), treatment with PI (4), treatment with CHX followed by LG (5), treatment with PI followed by LG (6), treatment with LG followed by CHX (7), treatment with LG followed by PI (8), treatment with a prepared mixture of CHX and LG (9), and treatment with a prepared mixture of PI and LG (10). CHX 0.1%, PI 0.5%, and LG were used. A volume of 0.5 mL for CHX and PI, and 1 mL for LG was inoculated onto the plates and distributed evenly using a seeding inoculation loop. The combinations of LG with CHX or PI (used in groups 9 and 10) were prepared as 1:1 mixtures and homogenized with a vortex; then 1mL was inoculated and distributed on the blood agar plates. Once treated, the plates were incubated at 37ºC with 5% CO2 for 24 h, after which the cfus were counted separately by two experts. All tests were performed in duplicate.

The statistical analyses were conducted using IBM-SPSS software for Windows (V 26) and the results are presented as mean and standard deviation or number and percentage as appropriate. The chi-square test was used to compare the studied variables among the three groups, with a level of significance set at 5% (alpha = 0.05) two tailed.

## Results

### MICs and MBCs

The average MIC values determined for all six tested microorganisms were 0.0004% for CHX (0.0001–0.0016%) and 0.2537% for PI (0.039–0.625%). The MBC values were found to be one or two dilutions higher than the MICs, with values of 0.0008% for CHX (0.0002–0.0032%) and 0.4425% for PI (0.156–0.625%). The highest MIC and MBC values were observed against *P. aeruginosa*, while *E. faecalis* resulted in the lowest PI MIC but second highest CHX MIC. (Table 1)

**Table 1:** Minimum inhibitory concentration and minimum bactericidal concentration results

	Povidone-iodine				Chlorhexidine			
	MIC^*^		MBC^†^		MIC^*^		MBC^†^	
	%	µg/ml	%	µg/ml	%	µg/ml	%	µg/ml
*P. aeruginosa*	0.625	6250	0.625	6250	0.0016	16	0.0032	32
*E. coli*	0.312	3120	0.312	3120	0.0002	2	0.0004	4
*S. agalactiae*	0.156	1560	0.625	6250	0.0001	1	0.0002	2
*S. aureus*	0.078	780	0.156	1560	0.0001	1	0.0002	2
*E. faecalis*	0.039	390	0.312	3120	0.0004	4	0.0004	4
*S. epidermidis*	0.312	3120	0.625	6250	0.0001	1	0.0004	4
Total, mean (range)	0.537 (0.039–0.625)	2537 (390–6250)	0.4425 (0.156–0.625)	4425 (1560–6250)	0.0004 (0.0001–0.0016)	4 (1–16)	0.0008 (0.0002–0.0032)	8 (2–32)

* MIC = Minimal Inhibitory Concentration

^†^ MBC = Minimum Bactericidal Concentrations

### Bacterial killing assays

The bacterial killing assay results indicate that there were no statistically significant differences between the observations of the two experts or between the duplicated tests (*p* = 0.08 and *p* = 0.36). Control group 1 (without treatments) and control group 2 (treatment only with LG) exhibited the expected number of colonies according to the initial inoculum, indicating that LG did not exert antibacterial activity. It was also observed that CHX was superior to PI (*p* = 0.001) in terms of reducing the number of cfus, with the highest reduction obtained when CHX was administered alone. A statistically significant difference (*p* = 0.001) was observed between CHX applications before and after the use of LG (groups 5 and 7), the most effective order of treatment being CHX followed by LG (group 5). This result suggests that CHX loses much of its bactericidal effect when administered after LG. PI was also more effective when applied prior to LG (group 6 vs. 8). Of the studied microorganisms, *E. faecalis* had the highest number of cfus, followed by *P. aeruginosa*. (Table 2)

**Table 2:** Mean number of colony-forming units for each bacterial strain by group*

	Group 1	Group 2	Group 3	Group 4	Group 5	Group 6	Group 7	Group 8	Group 9	Group 10
	Control	LG	CHX	PI	CHX then LG	PI then LG	LG then CHX	LG then PI	LG + CHX	LG + PI
*E. coli*	269 ± 82	268 ± 80	0	1 ± 1	4 ± 3	21 ± 13	3 ± 2	9 ± 5	0	5 ± 3
*E. faecalis*	647 ± 90	600 ± 101	0	0	0	0	39 ± 7	30 ± 8	51 ± 33	6 ± 3
*P. aeruginosa*	636 ± 162	526 ± 128	0	0	0	0	37 ± 15	28 ± 9	23 ± 13	3 ± 1
*S. agalactiae*	477 ± 140	518 ± 144	0	36 ± 20	0	0	0	54 ± 21	0	2 ± 1
*S. aureus*	310 ± 95	329 ± 102	0	7 ± 2	0	0	0	43 ± 9	0	6 ± 3
*S. epidermidis*	117 ± 36	139 ± 43	0	3 ± 2	0	1 ± 0	0	19 ± 11	0	10 ± 3

* Groups:

1. No treatment

2. Treatment with lidocaine gel (LG)

3. Treatment with aqueous chlorhexidine (CHX)

4. Treatment with povidone-iodine (PI)

5. Treatment with aqueous chlorhexidine (CHX) then lidocaine gel (LG).

6. Treatment with povidone-iodine (PI) then lidocaine gel (LG).

7. Treatment with lidocaine gel (LG) then aqueous chlorhexidine (CHX).

8. Treatment with lidocaine gel (LG) then povidone-iodine (PI).

9. Treatment with a prepared mixture of aqueous chlorhexidine (CHX) and lidocaine gel (LG).

10. Treatment with a prepared mixture of povidone-iodine (PI) and lidocaine gel (LG).

## Discussion

In this study, the in vitro efficacy of two different antiseptic solutions, administered alone and with a topical anesthetic, were evaluated against six bacteria. The ideal antiseptic for ocular use should have a rapid onset of action, be broad-spectrum bactericidal, and have low or no corneal toxicity. Widely used for antisepsis over several decades, PI possesses these properties, but it can cause significant postoperative ocular surface irritation [6, 22]. However, the optimal concentration for PI remains a topic of debate [6]. While lower concentrations of PI can release more free iodine, they also hold a smaller reservoir of iodine [23–25], which can be rapidly depleted in environments with high bacterial loads [26]. Although CHX 0.1% in an aqueous solution is not recommended for topical ocular use by the manufacturer, it has been applied in ophthalmic treatments for many years [6, 18]. The optimal concentration of CHX is not well-established. In clinical practice [6]. LG is often used as a topical anesthetic before cataract surgery. However, in vitro studies have indicated that the use of LG prior to PI reduces the effectiveness of antisepsis and increases microbial survivability [1, 27, 28].

A randomized clinical trial comparing CHX 0.1% with PI 5% for ocular antisepsis before IVI found that CHX 0.1% was associated with less ocular surface discomfort and corneal epitheliopathy than PI 5% during same-day bilateral IVIs. However, no significant differences in terms of positive microbial cultures or adverse events were observed between the two agents. It was therefore concluded that for some patients, CHX may be a better tolerated alternative for antimicrobial prophylaxis during IVIs compared to PI [29]. A study comparing the rates of endophthalmitis before and after transitioning from PI to CHX asepsis for intravitreal injections employed the same concentrations we did. The findings suggest that CHX 0.1% is a viable alternative to PI 5% [30]. Additionally, a randomized controlled trial comparing PI 0.6% with CHX 0.02% chlorhexidine demonstrated that CHX 0.02% significantly reduced the bacterial load on the ocular surface more effectively than PI 0.6%, without significant alterations in the taxonomic composition. CHX was also found to be better tolerated than PI [31]. There is no evidence for the superior effectiveness of PI vs. CHX and the best way to avoid endophthalmitis post-surgery has not been determined [32, 33].

A systematic review and meta-analysis highlighted the limitations of using MIC and MBC values as indicators of bacterial susceptibility and resistance to topical antiseptics. Instead, alternative methods that better reflect real-world clinical practice were proposed, such as models of topical application over a short period of time and experiments conducted on biofilm models [34]. However, the concentrations of both antiseptics used in clinical practice (CHX 0.1% and PI 5%) are well above the highest MICs and MBCs found in the present investigation.

In this laboratory-controlled study, we also tested combining an ocular surface antiseptic (PI or CHX) with a topical anesthetic (LG) in the same reagent, as a potential approach to improving prophylaxis before cataract surgery and reducing the risk of endophthalmitis following IVI. Our results support the hypothesis that the combination of PI or CHX with LG reduces bacterial growth to a level comparable to PI or CHX used alone [35]. However, it is important to note that the antiseptic must be applied separately prior to LG to achieve this effect. In agreement with our results, other studies have reported that both PI and CHX lose effectiveness if administered after LG [3, 6, 10, 27, 28, 36]. This suggests that LG may act as a physical barrier, preventing the antiseptic from penetrating and exerting an effect, or that a non-synergistic interaction occurs between the two chemicals. The most dramatic loss of effect was observed when CHX was mixed with LG before application, which might also point toward a chemical interaction. However, further studies are needed to test these hypotheses.

*E. faecalis* has been shown to be the bacterium most resistant to PI and CHX [37]. In our study, it was the species with the highest number of colonies in the bacterial killing assay, indicating that its eradication may be more challenging compared to the other studied microorganisms. However, the MIC and MBC values for *E. faecalis* were comparable to those of the other tested bacteria, suggesting that *E. faecalis* does not exhibit a higher level of resistance. Additionally, the concentrations of antiseptics used in the bacterial killing assay were found to be sufficient to inhibit *E. faecalis* growth.

## Conclusion

CHX 0.1% proved more effective than PI 5% in reducing bacterial growth. When used in combination with an anesthetic gel, both CHX and PI should be administered first for maximum effectiveness. Further chemical studies are needed to evaluate the interactions between lidocaine gel and CHX.

### Electronic supplementary material

Below is the link to the electronic supplementary material.

Supplementary Material 1

Supplementary Material 2

Supplementary Material 3

Supplementary Material 4

Supplementary Material 5

## Data Availability

No datasets were generated or analysed during the current study.

## Abbreviations

CHX: aqueous chlorhexidine
PI: povidone-iodine
LG: lidocaine gel
MICs: minimum inhibitory concentrations
MBCs: minimum bactericidal concentrations
Cfus: colonies forming units
IVI: intravitreal injection
PIE: postoperative intraocular endophthalmitis
