# Supplementary material for: Comparing the in vitro efficacy of chlorhexidine and povidone-iodine in the prevention of post-surgical endophthalmitis
Source: J Ophthalmic Inflamm Infect. 2024 May 23;14:20. doi: 10.1186/s12348-024-00404-2 (PMC11116284; doi:10.1186/s12348-024-00404-2)
Supplement: Supplementary file 1 — Supplementary Material 1 [file 12348_2024_404_MOESM1_ESM.pdf]

TECHNICAL DATA SHEET

1. **NAME OF THE MEDICINAL PRODUCT**

Clorxil 5 mg/ml cutaneous solution

2. **QUALITATIVE AND QUANTITATIVE COMPOSITION**

Each ml of solution contains: 5 mg of Chlorhexidine Digluconate.

For a full list of excipients, see section 6.1.

3. **PHARMACEUTICAL FORM**

Cutaneous solution

Clear and colorless solution

4. **CLINICAL PARTICULARS**

4.1 **Therapeutic Indications**

Disinfection of wounds, wound drainage, ulcers, ostomies, mucous membranes, and bladder catheterization.

4.2 **Posology and method of administration**

Posology:

Pediatric population:

Apply to the affected area once or twice a day.

Adults:

Apply to the affected area once or twice a day.

Method of administration:

For cutaneous use.

Use undiluted. Clean and dry the area to be treated before applying the medication. Apply directly to the affected area or on a gauze. Let it act and dry.

4.3 **Contraindications**

Hypersensitivity to chlorhexidine or to any of the excipients listed in section 6.1. Do not use in eyes or ears.

4.4 **Warnings and Special Precautions for Use**

External use on the skin only. Do not ingest. In case of accidental contact with eyes or ears, wash immediately with plenty of water. Although the absorption of chlorhexidine through the skin is minimal, the risk of systemic effects cannot be ruled out. These effects may be enhanced in case of repeated applications, use of the product over large areas, under occlusive dressings, on injured skin, and on mucous membranes. It should not be used for asepsis of puncture or injection areas, nor for the disinfection of surgical materials. It should not be used in case of deep and extensive wounds. Clothes that have been in contact with this medication

should not be washed with bleach or other hypochlorites, as this would cause a brown discoloration of the fabrics, but with domestic detergents based on sodium perborate.

Pediatric population:

The product should only be used under medical prescription in children under 30 months old. The use of chlorhexidine cutaneous solutions, whether alcoholic or aqueous, as a skin disinfectant prior to invasive procedures, has been associated with chemical burns in neonates. Based on reported cases and available publications, the risk appears greater in premature children, especially those born before week 32 of gestation and during the first 2 weeks of life.

Any soaked material, gauzes, or pajamas should be removed before starting the intervention. Do not use large amounts and avoid the solution accumulating in skin folds, under the patient, or soaking the sheets or other moist material in direct contact with the patient. When applying an occlusive dressing to areas previously exposed to Clorxil 5 mg/ml cutaneous solution, care should be taken that there is no excess product before placing the dressing.

4.5 **Interaction with Other Medicaments and Other Forms of Interaction**

In general: considering possible interferences (antagonism, inactivation, etc.), the simultaneous or successive use of antiseptics should be avoided, except with other cationic compounds. In particular: it should not be used in combination or after the application of cationic soaps, iodine, heavy metal salts, and acids. Its antiseptic activity is partially inhibited by organic products (serum, etc.) and phospholipids.

4.6 **Fertility, Pregnancy, and Lactation**

There are no or limited data available in humans, but reproduction studies in animals have shown no risk to the fetus (FDA category B).

It is unknown whether it is excreted in human breast milk, but no problems have been described in humans. However, the potential risk of systemic effects should be considered.

4.7 **Effects on the Ability to Drive and Use Machines**

The influence of Clorxil on the ability to drive and use machines is negligible or insignificant.

4.8 **Adverse Reactions**

- Risk of systemic effects (see section 4.4)
- Hypersensitivity and photosensitivity reactions.
- Traumatic injuries, poisonings, and complications of therapeutic procedures.
- Chemical burns in neonates (unknown frequency).

Notification of Suspected Adverse Reactions

It is important to report suspected adverse reactions following the authorization of the medication. This allows for continuous monitoring of the benefit/risk balance of the medication. Health professionals are encouraged to report suspected adverse reactions through the Spanish System of Pharmacovigilance for Human Use Medicines: https://www.notificaram.es.

4.9 **Overdose**

In case of accidental ingestion, proceed with gastric lavage and protection of the gastric mucosa. Cases of hemolysis have been described following the ingestion of chlorhexidine. In cases of hemolysis, a blood transfusion may be necessary.

5. **PHARMACOLOGICAL PROPERTIES**

5.1 **Pharmacodynamic Properties**

Pharmacotherapeutic group: Antiseptics and disinfectants. Biguanides and amidines: Chlorhexidine. ATC code: D08AC02.

Chlorhexidine is a cationic biguanide compound used as a topical antiseptic active against a broad spectrum of Gram-positive and Gram-negative microorganisms. It is more effective against Gram-positive than Gram-negative bacteria. Generally, chlorhexidine is not considered active against acid-alcohol-resistant bacteria, fungi, spores, and viruses. Chlorhexidine reacts with the anionic groups on the bacterial surface, altering its permeability.

5.2 **Pharmacokinetic Properties**

The absorption of chlorhexidine through the skin is minimal. In the event of systemic absorption, elimination is carried out via the bile or at the renal level, without any preceding metabolite.

Digestive absorption is virtually nil (around 99% of the ingested dose is excreted unchanged in the feces).

The antibacterial activity of chlorhexidine on the skin persists for several hours after its application.

5.3 **Preclinical Safety Data**

Toxicity studies conducted in experimental animals with chlorhexidine have shown that its toxicity is practically nil under the proposed conditions. Studies on carcinogenesis, mutagenesis, and teratogenesis have not shown signs of such activity.

6. **PHARMACEUTICAL PARTICULARS**

6.1 **List of Excipients**

Purified water

6.2 **Incompatibilities**

This medicine is incompatible with anionic derivatives (soaps, etc.) as chlorhexidine behaves as a cationic; therefore, this active ingredient precipitates at pH above 8 in the presence of numerous anions.

6.3 **Period of Validity**

2 years.

Discard 3 months after opening the package.

6.4 **Special Precautions for Storage**

Does not require special storage conditions.

6.5 **Nature and Content of the Container**

Clorxil 5mg/ml cutaneous solution is presented in:

White HDPE bottles with HDPE/LDPE cap, in the following presentations:

Single packages:

1 bottle of 100ml

1 bottle of 250ml

Clinical packages:

100 bottles of 10ml

50 bottles of 50ml

50 bottles of 100ml

50 bottles of 250ml

20 bottles of 500ml

Some package sizes may only be marketed.

6.6 **Special Precautions for Disposal and Other Handling**

The disposal of unused medicine and all materials that have been in contact with it should be carried out according to local regulations.

7. **MARKETING AUTHORIZATION HOLDER**

LABORATORIOS BOHM, S.A.
C/ Molinaseca 23-25, Polígono Industrial Cobo Calleja,
28947 Fuenlabrada (Madrid),
Spain
Phone: 91 642 18 18

8. **MARKETING AUTHORIZATION NUMBER(S)**

9. **DATE OF FIRST AUTHORIZATION/RENEWAL OF THE AUTHORIZATION** September 2022

10. **DATE OF REVISION OF THE TEXT**

September 2022

Detailed and updated information on this medicine is available on the website of the Spanish Agency for Medicines and Health Products (AEMPS) http://www.aemps.gob.es.
