# Supplementary material for: Comparing the in vitro efficacy of chlorhexidine and povidone-iodine in the prevention of post-surgical endophthalmitis
Source: J Ophthalmic Inflamm Infect. 2024 May 23;14:20. doi: 10.1186/s12348-024-00404-2 (PMC11116284; doi:10.1186/s12348-024-00404-2)
Supplement: Supplementary file 2 — Supplementary Material 2 [file 12348_2024_404_MOESM2_ESM.pdf]

TECHNICAL DATA SHEET

1. **NAME OF THE MEDICINAL PRODUCT**

Curadona 100 mg/ml cutaneous solution

2. **QUALITATIVE AND QUANTITATIVE COMPOSITION**

Each milliliter of solution contains:

Povidone-Iodine.......................................100 mg

For the complete list of excipients, see section 6.1.

3. **PHARMACEUTICAL FORM**

Brown cutaneous solution.

4. **CLINICAL PARTICULARS**

4.1 **Therapeutic Indications**

General skin antiseptic for:

- Small wounds and superficial cuts
- Minor burns or abrasions

In clinical settings:

- General antiseptic and disinfectant. In hospital settings, indicated as an antiseptic for the surgical field, puncture areas, wounds, burns, and surgical materials. Microbial and mycotic dermatitis. Disinfection by irrigation of septic body areas: peritoneum, pleura, bones.

4.2 **Posology and method of administration**

Apply a small amount directly to the affected area, 2 to 3 times a day.

Cutaneous use:

- Wash and dry the affected area before applying the product.

4.3 **Contraindications**

Do not administer the product in the following cases:

- Hypersensitivity to iodinated products or medicines or to any of the excipients in this medicine.
- Neonates (0 to 1 month).

4.4 **Warnings and Special Precautions for Use**

- Regular or prolonged use of this product should be avoided, especially in patients with:
    - Burns affecting more than 20% of the body surface
    - Large or open wounds

- Liver failure
- Kidney failure
- Thyroid disorders
- Established treatment with lithium

In cases where prolonged use of povidone-iodine is necessary or must be applied to burns or extensive areas of the skin, thyroid function tests should be performed.

- Do not heat the product before use.
- Avoid contact with eyes, ears, and other mucous membranes.

4.5 **Interactions with Other Medicaments and Other Forms of Interaction**

Do not apply povidone-iodine at the same time as products containing:

- **Mercurial derivatives**, as they react with iodine to form compounds that are irritating.
- **Sodium thiosulfate**, as both products are inactivated before they can act.

Prolonged use of povidone-iodine should be avoided in patients undergoing simultaneous therapy with lithium.

<u>Interferences with diagnostic tests</u>:

- Absorption through intact or damaged skin of the iodine contained in povidone-iodine can interfere with the results of thyroid function tests. False positives may occur in several types of tests for the detection of occult blood in feces or urine.

4.6 **Fertility, Pregnancy, and Lactation**

Continued use of povidone-iodine on extensive areas of the skin should be avoided in pregnant or lactating women, as the absorbed iodine can cross the placental barrier and be excreted in breast milk, potentially causing transient hypothyroidism in the fetus or nursing infant, especially if used during the perinatal period by women living in areas with nutritional iodine deficiency.

4.7 **Effects on the Ability to Drive and Use Machines**

No effects on the ability to drive and use machines have been described.

4.8 **Adverse Reactions**

During the period of use of povidone-iodine, the following adverse reactions have been reported, although their frequency has not been accurately established: rarely local skin reactions such as local irritation, itching, or burning.

The application of povidone-iodine on extensive wounds, burns, or for a prolonged time, may produce adverse systemic effects, such as metabolic acidosis, hypernatremia, and disorders of renal, hepatic, and thyroid function (especially in children).

In the event of adverse reactions, they should be reported to Pharmacovigilance systems, and if necessary, treatment should be discontinued.

4.9 **Overdose**

With excessive use of povidone-iodine, skin irritation may rarely occur. If this occurs, treatment should be discontinued, the affected area washed thoroughly with water, and topical anti-inflammatory treatments (including corticosteroids) applied.

Continued and prolonged exposure to iodine may cause goiter, hypothyroidism, or hyperthyroidism.

In cases of accidental ingestion of large amounts of povidone-iodine, symptoms may include abdominal pain, diarrhea, fever, nausea, vomiting, metabolic acidosis, and hypernatremia, as well as alterations in renal, hepatic, and thyroid functions. In these cases, if the patient is conscious, they should drink milk every 15 minutes to alleviate gastric irritation. Additionally, to absorb any remaining povidone-iodine, a starch solution should be administered, prepared by adding 15 mg of cornstarch or 15 mg of flour to 500 ml of water. If the patient suffers from esophageal damage, washing or emesis should not be performed.

5. PHARMACOLOGICAL PROPERTIES

5.1 **Pharmacodynamic Properties**

Pharmacotherapeutic group: Dermatologicals. Antiseptics and disinfectants: Iodine products. Povidone-iodine.

ATC code: D08AG02.

Povidone-iodine is a broad-spectrum antiseptic with bactericidal, fungicidal, virucidal, antiprotozoal, and sporicidal activity. It is a molecular complex of iodine with povidone. The complex, as such, lacks activity until iodine is released, which is the actual agent responsible for the antiseptic effect. Dilution increases the iodine release process.

Iodine acts through oxidation-reduction reactions, altering many biologically important molecules such as glucose, starch, glycols, lipids, amino acids, proteins, etc. The bactericidal action is rapid (within seconds or minutes). In this process, iodine is transformed into iodide, which is microbiologically inactive. Iodine has the broadest known antimicrobial spectrum. No emergence of resistant microbial strains has been detected.

Its microbicidal activity is maintained in the presence of blood, pus, serum, and necrotic tissue, thus maintaining its activity in infections in septic body cavities such as the pleura, peritoneum, bone, and bladder.

The antimicrobial activity of povidone-iodine is affected by the pH of the medium, being optimal in acidic conditions.

5.2 **Pharmacokinetic Properties**

In cutaneous use, the absorption of iodine contained in povidone-iodine through the skin is minimal, and it is excreted unchanged in the urine.

The absorption of iodine is greater in vaginal application and on damaged skin.

5.3 **Preclinical Safety Data**

In various acute toxicity studies, LD50 values for povidone-iodine have been determined. Orally, values have been found between 210 mg of iodine/kg in mice and 1300 mg of iodine/kg in rats.

Intraperitoneally, the interspecies threshold value (mouse, rat, dog) is close to 25 mg of iodine/kg.

The gradual release of free iodine from povidone-iodine explains its lower toxicity compared to iodine in cutaneous use.

6. PHARMACEUTICAL PARTICULARS

6.1 **List of Excipients**

- Glycerol
- Polyoxiethylene alkyl ether
- Disodium phosphate
- Citric acid
- Sodium hydroxide
- Purified water

6.2 **Incompatibilities**

Do not apply povidone-iodine together with mercurial derivatives, due to the risk of forming caustic compounds.

As iodine is an oxidant, it should not be administered together with substances with which it has chemical incompatibility, such as organic compounds which may undergo oxidation and thus become inactive upon contact with povidone-iodine.

Iodine is inactivated by sodium thiosulfate, and by the action of light, heat, and alkaline pH.

6.3 **Period of Validity**

The validity period is 3 years.

6.4 **Special Precautions for Storage**

No special storage conditions required.

6.5 **Nature and Contents of the Container**

Containers made of polypropylene of 30 and 60 ml and high-density polyethylene of 250 and 500 ml.

Clinical packages of 10 bottles of 250 and 500 ml. 25 bottles of 125 ml.

6.6 **Special Precautions for Disposal**

The disposal of unused medicine and all materials that have been in contact with it should be carried out in accordance with local regulations or returned to the pharmacy.

7. MARKETING AUTHORIZATION HOLDER

LAINCO, S.A.

Avda. Bizet, 8-12

08191 Rubí (Barcelona)

SPAIN

8. MARKETING AUTHORIZATION NUMBER(S)

63.031

9. DATE OF FIRST AUTHORIZATION/RENEWAL OF THE AUTHORIZATION

May 8, 2000

10. DATE OF REVISION OF THE TEXT

August 2009
