# Supplementary material for: Comparing the in vitro efficacy of chlorhexidine and povidone-iodine in the prevention of post-surgical endophthalmitis
Source: J Ophthalmic Inflamm Infect. 2024 May 23;14:20. doi: 10.1186/s12348-024-00404-2 (PMC11116284; doi:10.1186/s12348-024-00404-2)
Supplement: Supplementary file 3 — Supplementary Material 3 [file 12348_2024_404_MOESM3_ESM.pdf]

FICHA TÉCNICA

1. NOMBRE DEL MEDICAMENTO

Curadona 100 mg/ml solución cutánea

2. COMPOSICIÓN CUALITATIVA Y CUANTITATIVA

Cada mililitro de solución contiene:
Povidona (DOE) iodada.......................................100 mg

Para consultar la lista completa de excipientes, ver sección 6.1.

3. FORMA FARMACÉUTICA

Solución cutánea de color marrón.

4. DATOS CLÍNICOS

4.1 Indicaciones terapéuticas

Antiséptico de la piel de uso general en: pequeñas heridas y cortes superficiales, quemaduras leves o rozaduras.
<u>En envases clínicos:</u>
Antiséptico y desinfectante de uso general. En el ámbito hospitalario, indicado como antiséptico del campo operatorio, zonas de punción, heridas, quemaduras y material quirúrgico. Dermatitis microbianas y micósicas. Desinfección por irrigación de zonas sépticas corporales: peritoneo, pleura, huesos.

4.2 Posología y forma de administración

Aplicar una pequeña cantidad directamente sobre la zona afectada, de 2 a 3 veces al día.
Uso cutáneo
Lavar y secar la zona afectada antes de aplicar el producto.

4.3 Contraindicaciones

No administrar el producto en los siguientes casos:

- Hipersensibilidad a productos o medicamentos iodados o a alguno de los excipientes de este medicamento.
- Neonatos (0 a 1 mes).

4.4 Advertencias y precauciones especiales de empleo

- Debe evitarse el uso regular o prolongado de este producto, especialmente en pacientes con:

- quemaduras que afecten a más del 20% de la superficie corporal
- heridas grandes o abiertas
- insuficiencia hepática
- fallo renal
- trastornos tiroideos
- tratamiento establecido con litio.

En los casos en que resulte necesaria una utilización prolongada de povidona iodada o deba ser aplicada en quemaduras o en áreas extensas de la piel, deberán realizarse pruebas de la función tiroidea.
-No calentar el producto antes de su utilización.
-Evitar el contacto con los ojos, oídos y otras mucosas.

MINISTERIO DE SANIDAD, POLITICA SOCIAL E IGUALDAD
Agencia española de medicamentos y productos sanitarios

-La utilización en niños menores de 30 meses, si fuese indispensable, se limitará a una aplicación breve y poco extensa, seguida de un enjuague con agua estéril.

4.5 Interacción con otros medicamentos y otras formas de interacción

No aplicar povidona iodada al mismo tiempo que otros productos que contengan:
- **derivados mercuriales**, ya que éstos reaccionan con el iodo formando compuestos que son irritantes.
- **tiosulfato de sodio**, ya que se inactivan ambos productos antes de ejercer su acción

Se debe evitar el uso prolongado de povidona iodada en pacientes que se encuentren en terapia simultánea con litio.

Interferencias con pruebas de diagnóstico:
La absorción, a través de la piel intacta o dañada, del iodo contenido en la povidona iodada puede interferir en los resultados de las pruebas de la función tiroidea. Pueden obtenerse falsos positivos en varios tipos de pruebas para la detección de sangre oculta en heces u orina.

4.6 Fertilidad, embarazo y lactancia

Debe evitarse el uso continuado de povidona iodada en áreas extensas de la piel, en mujeres embarazadas o en periodo de lactancia, ya que el iodo absorbido puede atravesar la barrera placentaria así como excretarse por la leche materna, por lo que podría llegar a producir hipotiroidismo transitorio en el feto o en el lactante, especialmente si el uso tiene lugar durante el periodo perinatal de mujeres que residen en zonas con déficit nutricional de iodo.

4.7 Efectos sobre la capacidad para conducir y utilizar máquinas

No se han descrito efectos sobre la capacidad para conducir y utilizar máquinas.

4.8 Reacciones adversas

Durante el periodo de utilización de la povidona iodada, se han notificado las siguientes reacciones adversas, cuya frecuencia no se ha podido establecer con exactitud: raramente reacciones cutáneas locales, como irritación local, prurito o quemazón.
La aplicación de povidona iodada sobre heridas extensas, quemaduras o durante tiempo prolongado, puede producir efectos sistémicos adversos, tales como acidosis metabólica, hipernatremia y trastornos de la función renal, hepática y tiroidea (especialmente en niños).
En caso de observarse la aparición de reacciones adversas, deben notificarse a los sistemas de Farmacovigilancia y, en caso necesario, suspender el tratamiento.

4.9 Sobredosis

Con el uso excesivo de povidona iodada, raramente se puede producir irritación de la piel. Pero si esto ocurriera, se debe suspender el tratamiento, lavar la zona afectada con abundante agua y aplicar antiinflamatorios de uso tópico (incluidos los corticoides).

Una exposición continua y prolongada al iodo puede producir bocio, hipotiroidismo o hipertiroidismo.
En casos de ingestión accidental de grandes cantidades de povidona iodada, pueden producirse: dolor abdominal, diarrea, fiebre, náuseas, vómitos, acidosis metabólica e hipernatremia, así como una alteración de las funciones renales, hepáticas y tiroideas. En estos casos, si el paciente está consciente, deberá ingerir leche cada 15 minutos para aliviar la irritación gástrica. Además, con el fin de absorber la povidona iodada remanente, habrá que administrarle una solución de almidón, preparada adicionando 15 mg de almidón de maíz o 15 mg de harina sobre 500 ml de agua. Si el paciente sufre daño esofágico no se podrá efectuar el lavado o la emesis.

MINISTERIO DE SANIDAD, POLITICA SOCIAL E IGUALDAD
Agencia española de medicamentos y productos sanitarios

Se puede recurrir a la aplicación de otras medidas de apoyo para el mantenimiento de las funciones vitales, como la administración de oxígeno para mantener la respiración y la administración de antihistamínicos, epinefrina o corticosteroides para el tratamiento de la anafilaxia.

5. PROPIEDADES FARMACOLÓGICAS

5.1 Propiedades farmacodinámicas

Grupo farmacoterapéutico: dermatológicos. Antisépticos y desinfectantes: productos de iodo. Povidona iodada
Código ATC: D08AG02
La povidona iodada es un antiséptico de amplio espectro, con actividad antibacteriana (bactericida), antifúngica (fungicida), antiviral, antiprotozoaria y esporicida. Se trata de un complejo molecular de iodo con povidona. El complejo, como tal, carece de actividad hasta que se va liberando el iodo, verdadero responsable del efecto antiséptico. La dilución incrementa el proceso de liberación de iodo.

El iodo actúa mediante reacciones de óxido-reducción, alterando muchas moléculas biológicamente importantes, como glucosa, almidón, glicoles, lípidos, aminoácidos, proteínas, etc. La acción bactericida se manifiesta rápidamente (en unos segundos o minutos). En este proceso, el iodo se transforma en ioduro, que es microbiológicamente inactivo. El iodo tiene el espectro antimicrobiano más amplio conocido. No se ha detectado la aparición de cepas microbianas resistentes.
Su actividad microbicida se mantiene en presencia de sangre, pus, suero y en tejido necrótico por lo que mantiene su actividad en caso de infecciones en cavidades sépticas corporales, como en pleura, peritoneo, hueso y vejiga.

La actividad antimicrobiana de la povidona iodada se ve afectada por el pH del medio, siendo óptima en condiciones ácidas.

5.2 Propiedades farmacocinéticas

En el uso cutáneo, la absorción a través de la piel del iodo que contiene la povidone iodada resulta mínima, siendo eliminado de forma inalterada por la orina.
La absorción del iodo es mayor en la aplicación vaginal y en piel lesionada.

5.3 Datos preclínicos sobre seguridad

En diferentes estudios de toxicidad aguda se han determinado los valores de DL_{50} para la povidona iodada. Por vía oral, se han encontrado valores entre 210 mg de iodo/kg en ratón y 1300 mg de iodo/kg en rata. Por vía intraperitoneal, el valor umbral interespecie (ratón, rata, perro) se encuentra próximo a 25 mg de iodo/kg.
La liberación gradual del iodo libre de la povidona iodada explica la menor toxicidad de ésta frente al iodo en el uso cutáneo.

6 . DATOS FARMACÉUTICOS

6.1 Lista de excipientes

- Glicerol
- Alquileter polioxietileno
- Fosfato de disodio
- Ácido cítrico
- Hidróxido de sodio
- Agua purificada

MINISTERIO DE
SANIDAD, POLITICA
SOCIAL E IGUALDAD
Agencia española de
medicamentos y
productos sanitarios

6.2 Incompatibilidades

No aplicar povidona iodada conjuntamente con derivados mercuriales, por el riesgo de que se produzcan compuestos cáusticos.
Al ser el iodo un oxidante, no se debe administrar conjuntamente con aquellas sustancias con las que presente incompatibilidad química como los compuestos orgánicos que en contacto con povidona iodada pueden sufrir oxidación y en consecuencia, transformarse en sustancias inactivas.

El iodo es inactivado por el tiosulfato de sodio, y por la acción de la luz, el calor y el pH alcalino.

6.3 Periodo de validez

El periodo de validez es de 3 años.

6.4 Precauciones especiales de conservación

No precisa condiciones especiales de conservación.

6.5 Naturaleza y contenido del envase

Envases de polipropileno de 30 y 60 ml y de polietileno de alta densidad de 250 y 500 ml.
Envases clínicos de 10 botellas de 250 y 500 ml. 25 frascos de 125 ml.

6.6 Precauciones especiales de eliminación

La eliminación del medicamento no utilizado y de todos los materiales que hayan estado en contacto con él se realizará de acuerdo con las normativas locales, o se procederá a su devolución a la farmacia.

7. TITULAR DE LA AUTORIZACIÓN DE COMERCIALIZACIÓN

LAINCO, S.A.
Avda. Bizet, 8-12
08191 Rubí (Barcelona)
ESPAÑA

8. NÚMERO(S) DE AUTORIZACIÓN DE COMERCIALIZACIÓN

63.031

9. FECHA DE LA PRIMERA AUTORIZACIÓN/ RENOVACIÓN DE LA AUTORIZACIÓN

8.5.00

10. FECHA DE LA REVISIÓN DEL TEXTO

Agosto 2009

MINISTERIO DE
SANIDAD, POLITICA
SOCIAL E IGUALDAD
Agencia española de
medicamentos y
productos sanitarios
