# Supplementary material for: Comparing the in vitro efficacy of chlorhexidine and povidone-iodine in the prevention of post-surgical endophthalmitis
Source: J Ophthalmic Inflamm Infect. 2024 May 23;14:20. doi: 10.1186/s12348-024-00404-2 (PMC11116284; doi:10.1186/s12348-024-00404-2)
Supplement: Supplementary file 4 — Supplementary Material 4 [file 12348_2024_404_MOESM4_ESM.pdf]

SUMMARY OF PRODUCT CHARACTERISTICS,
LABELLING AND PACKAGE LEAFLET

SUMMARY OF PRODUCT CHARACTERISTICS

1. NAME OF THE MEDICINAL PRODUCT

Ophtesic 20 mg/g, eye gel in a single dose container

2. QUALITATIVE AND QUANTITATIVE COMPOSITION

1 g of gel contains lidocaine hydrochloride equivalent to 20 mg of lidocaine hydrochloride anhydrous.

For the full list of excipients, see section 6.1.

3. PHARMACEUTICAL FORM

Eye gel. Homogenous, clear transparent gel.

4. CLINICAL PARTICULARS

4.1 Therapeutic indications

Topical anaesthesia during ophthalmic procedures.

4.2 Posology and method of administration

Ophtesic should be used for topical application of gel only, and is not intended for use by another route of administration.

Posology

Cover the ocular surface, and eye adnexa if needed, with the gel in the area of the planned ophthalmic procedure, 5 minutes before the procedure, after disinfection.

1 g spread at the surface of the eye is usually enough to get an initial anaesthesia. Additional quantity can be used if needed depending on the size of the eye or the duration of the procedure.

1 gram corresponds roughly to one third of a tube.

Do not exceed one tube per eye or per single procedure.

The gel may be reapplied to maintain anaesthetic effect.

Paediatric population
The safety and efficacy of this medicine have not been established in children.

Method of administration

In case of surgery, scrupulous disinfection must be done before spreading the lidocaine gel.
If needed, the gel can be rinsed after 5 minutes' contact with surface of the eye.

4.3 Contraindications

Hypersensitivity to the active substance, to other amide-type local anesthetic or to any of the excipients listed in section 6.1.

4.4 Special warnings and precautions for use

For ocular use.

Corneal opacification: prolonged use of a topical ocular anaesthetic may produce permanent corneal opacification and ulceration with accompanying visual loss.

Ophtesic is intended for single administration and should be discarded immediately after use.

4.5 Interaction with other medicinal products and other forms of interaction

No interaction studies with other medicinal products have been performed.

4.6 Fertility, pregnancy and lactation

<u>Pregnancy</u>
There is a limited amount of data (less than 300 pregnancy outcomes) for the topical use of lidocaine 2% gel in pregnant women. Animal studies do not indicate direct or indirect harmful effects with respect to reproductive toxicity (see section 5.3). No effects during pregnancy are anticipated, since systemic exposure to lidocaine is negligible. Ophtesic can be used during pregnancy.

<u>Lactation</u>
Lidocaine is secreted in human milk. No effects on the breastfed new-born/infant/are anticipated since systemic exposure of the breastfeeding woman to lidocaine is negligible.

<u>Fertility</u>
Studies have not been performed to evaluate the effect of topical ocular administration of lidocaine on human fertility.

4.7 Effects on ability to drive and use machines

Patients should be advised not to drive or operate hazardous machinery until normal vision is restored.

4.8 Undesirable effects

The undesirable effects are listed by MedDRA System Organ Classes.
Assessment of undesirable effects is based on the following frequency groupings:
Very common: ≥1/10
Common: ≥1/100 to <1/10
Uncommon: ≥1/1,000 to <1/100
Rare: ≥1/10,000 to <1/1,000
Very rare: <1/10,000
Not known: cannot be estimated from the available data

**Systeme Organ class**	**Undesirable effect**	**frequency**
Eye disorders	conjunctival hyperemia	Not known
	corneal epithelial changes	Not known
	eye burning sensation	Not known
	corneal punctuate keratitis	Not known

	corneal oedema	Not known
Nervous system disorders	headache	Not known

The mostly reported adverse reactions are conjunctival hyperemia, corneal epithelial changes, headache and burning upon instillation.
Corneal disorders such as superficial punctuate keratitis or oedema may be observed following short-term application of ophthalmic gel for topical anaesthesia.
The cornea may be damaged by prolonged application of anaesthetic eye gel.

Reporting of suspected adverse reactions
Reporting suspected adverse reactions after authorisation of the medicinal product is important. It allows continued monitoring of the benefit/risk balance of the medicinal product. Healthcare professionals are asked to report any suspected adverse reactions via the national reporting system listed in Appendix V.*

4.9 Overdose

Prolonged use of a topical ocular anaesthetic may produce permanent corneal opacification and ulceration with accompanying visual loss.

Acute emergencies from local anaesthetics are generally related to high plasma levels encountered during therapeutic use of local anaesthetics or to unintended subarachnoid injection of local anaesthetic solution.

However, topical application of Ophtesic is not expected to result in systemic exposure.

5. PHARMACOLOGICAL PROPERTIES

5.1 Pharmacodynamic properties

Pharmacotherapeutic group: Ophthalmologicals, Local anesthetics, ATC code: S01HA07

Lidocaine is an established topical anaesthetic from the amide group that stabilizes the neuronal membrane by inhibiting the ionic fluxes required for the initiation and conduction of impulses, thereby effecting local anaesthetic action. Anaesthesia generally occurs between 20 seconds to 1 minute, peaks between 3 to 5 minutes, and persists for 5 to 30 minutes.

5.2 Pharmacokinetic properties

Absorption
Lidocaine may be absorbed following topical administration to mucous membranes. Its rate and extent of absorption being dependent upon the concentration and total dose administered , the specific site of application, and duration of exposure.
Following ocular application of lidocaine, systemic exposure is very low.

Distribution
The plasma protein binding of lidocaine is dependent on the drug concentration, and the fraction bound decreases with increasing concentration. At concentrations of 1 to 4 microgram of free base per ml, 60 to 80 percent of lidocaine is protein-bound. Binding is also dependent on the plasma concentration of the alpha-1-acid glycoprotein.

Biotransformation

Lidocaine is metabolized rapidly by the liver, and metabolites and unchanged drug are excreted by the kidneys. Biotransformation includes oxidative N-dealkylation, ring hydroxylation, cleavage of the amide linkage, and conjugation.
N-dealkylation, a major pathway of biotransformation, yields the metabolites monoethylglycinexylidide and glycinexylidide. The pharmacological/toxicological actions of these metabolites are similar to, but less potent than, those of lidocaine. Approximately 90% of lidocaine administered is excreted in the form of various metabolites, and less than 10% is excreted unchanged. The primary metabolite in urine is a conjugate of 4-hydroxy-2, 6-dimethylaniline.

Elimination

The elimination half-life of lidocaine following an intravenous bolus injection is typically 1.5 to 2.0 hours. Because of the rate at which lidocaine is metabolized, any condition that affects liver function may alter lidocaine kinetics. The half-life may be prolonged two-fold or more in patients with liver dysfunction. Renal dysfunction does not affect lidocaine kinetics but may increase the accumulation of metabolites.

5.3 Preclinical safety data

In conventional animal studies, CNS and cardiovascular system toxicity was seen only after high doses of lidocaine. No drug related adverse effects were seen in reproduction toxicity studies. Carcinogenicity studies have not been performed with lidocaine, due to the limited duration of therapeutic use.

Genotoxicity tests with lidocaine showed no evidence of mutagenic potential. A metabolite of lidocaine, 2-6- dimethylaniline, showed weak evidence of activity in some genotoxicity tests.
The metabolite 2-6- dimethylaniline, has been shown to have carcinogenicity potential in preclinical toxicological studies evaluating chronic exposure. Risk assessments comparing the calculated maximum human exposure from intermittent use of lidocaine, with the exposure used in preclinical studies, indicate a wide margin of safety for clinical use.

6. PHARMACEUTICAL PARTICULARS

6.1 List of excipients

Hypromellose (E464), type 2910
Sodium hydroxide (E524)
Hydrochloric acid (E507) (for pH adjustment)
Water for injections

6.2 Incompatibilities

In the absence of compatibility studies, this medicinal product must not be mixed with other medicinal products.

6.3 Shelf life

Unopened: 3 years
Shelf-life after first opening: discard immediately after use.

6.4 Special precautions for storage

Do not store above 25 °C .

6.5 Nature and contents of container

Gel in aluminium tube epoxy coated and polypropylene nozzle, overwraped in a polypropylene/kraft paper blister.

The nozzle is inside the blister and should be attached to the tube before administration of the product.

The inside of the blister is sterile until opening.

One tube contains 3.5 g

Box of 1, 20, or 100 tubes.

6.6 Special precautions for disposal

No special requirements.
Any unused medicinal product or waste material should be disposed of in accordance with local requirements.

7. MARKETING AUTHORISATION HOLDER

LDD
93, rue Jean Jaurès
92800 Puteaux
France

8. MARKETING AUTHORISATION NUMBER(S)

1 tube
20 tubes
100 tubes

9. DATE OF FIRST AUTHORISATION/RENEWAL OF THE AUTHORISATION

<Date of first authorisation: {DD month YYYY}>

10. DATE OF REVISION OF THE TEXT

12/2021

LABELLING

PARTICULARS TO APPEAR ON THE OUTER PACKAGING

CARTON BOX

1. NAME OF THE MEDICINAL PRODUCT

Ophtesic 20 mg/g, eye gel in a single dose container.

Lidocaine hydrochloride

2. STATEMENT OF ACTIVE SUBSTANCE(S)

1 g of gel contains 20 mg of lidocaine hydrochloride (anhydrous).

3. LIST OF EXCIPIENTS

Hypromellose (E464) type 2910, sodium hydroxide (E524), hydrochloric acid (E507) (for pH adjustment, water for injections).

4. PHARMACEUTICAL FORM AND CONTENTS

Eye gel
3.5g
Box of 1, 20, 100 tube(s)

5. METHOD AND ROUTE(S) OF ADMINISTRATION

For ocular use.
Read the package leaflet before use.
Discard after use.

6. SPECIAL WARNING THAT THE MEDICINAL PRODUCT MUST BE STORED OUT OF THE SIGHT AND REACH OF CHILDREN

Keep out of the sight and reach of children.
Do not store above 25°C

7. OTHER SPECIAL WARNING(S), IF NECESSARY

Sterile

8. EXPIRY DATE

Exp: xx/xxxx
Discard after first use.

9. SPECIAL STORAGE CONDITIONS

10. SPECIAL PRECAUTIONS FOR DISPOSAL OF UNUSED MEDICINAL PRODUCTS OR WASTE MATERIALS DERIVED FROM SUCH MEDICINAL PRODUCTS, IF APPROPRIATE

11. NAME AND ADDRESS OF THE MARKETING AUTHORISATION HOLDER

LDD
93, rue Jean Jaurès
92800 Puteaux
France

12. MARKETING AUTHORISATION NUMBER(S)

13. BATCH NUMBER

Batch XX

14. GENERAL CLASSIFICATION FOR SUPPLY

Prescription only

15. INSTRUCTIONS ON USE

16. INFORMATION IN BRAILLE

None (professional use only).

Justification for not including Braille accepted.

17. UNIQUE IDENTIFIER – 2D BARCODE

2D barcode carrying the unique identifier included.

18. UNIQUE IDENTIFIER – HUMAN READABLE DATA

PC: {number}
SN: {number}
NN: {number}

MINIMUM PARTICULARS TO APPEAR ON BLISTERS OR STRIPS

STERILE BLISTER

1. NAME OF THE MEDICINAL PRODUCT

Ophtesic 20 mg/g, eye gel in a single dose container

Lidocaine hydrochloride

2. NAME OF THE MARKETING AUTHORISATION HOLDER

LDD
93, rue Jean Jaurès
92800 Puteaux
France

3. EXPIRY DATE

EXP: xx/xxxx

4. BATCH NUMBER

Batch XX

5. OTHER

Sterile blister to be opened just before use.
Do not store above 25°C.
Discard after use

MINIMUM PARTICULARS TO APPEAR ON SMALL IMMEDIATE PACKAGING UNITS

ALUMINIUM TUBE

Mentions limited due to the small size of the tube (4x1 cm)

1. NAME OF THE MEDICINAL PRODUCT AND ROUTE(S) OF ADMINISTRATION

Ophtesic 20 mg/g, eye gel in a single dose container

Lidocaine hydrochloride

For ocular use

2. METHOD OF ADMINISTRATION

Not applicable.

3. EXPIRY DATE

EXP: xx/xxxx

4. BATCH NUMBER

Batch XX

5. CONTENTS BY WEIGHT, BY VOLUME OR BY UNIT

3.5 g

6. OTHER

Discard after use

PACKAGE LEAFLET

Package leaflet: Information for the patient

Ophtesic20 mg/g, eye gel in a single dose container
Lidocaine hydrochloride

Read all of this leaflet carefully before you start using this medicine because it contains important information for you.
Always use this medicine exactly as described in this leaflet or as your doctor told you.
- Keep this leaflet. You may need to read it again.
- If you have any further questions, ask your doctor.
- If you get any side effects, talk to your doctor. This includes any possible side effects not listed in this leaflet. See section 4.

What is in this leaflet

1. What Ophtesic is and what it is used for
2. What you need to know before you use Ophtesic
3. How to use Ophtesic
4. Possible side effects
5. How to store Ophtesic
6. Contents of the pack and other information

1. What Ophtesic is and what it is used for

Ophtesic is an anaesthetic eye gel used during ophthalmic procedures.

Ophtesic is used to produce a temporary loss of feeling on your eye before and during certain types of procedures done by your doctor.

Ophtesic should start to work within 5 minutes after your doctor apply it.

2. What you need to know before you use Ophtesic

Do not use Ophtesic:
- If you are allergic to lidocaine, to any other local anaesthetic, or any of the other ingredients of this medicine (listed in section 6).

Warnings and precautions
- For ocular use only.
- The prolonged use of this type of eye anaesthetic may induce opacification of your cornea.
- Ophtesic 20 mg/g, eye gel in a single dose container is intended for single administration and should be discarded immediately after use.

Other medicines and Ophtesic
Tell your doctor if you are taking, have recently taken or might take any other medicines.

Pregnancy, breast-feeding and fertility
If you are pregnant or breast-feeding, think you may be pregnant or are planning to have a baby, ask your doctor for advice before using this medicine.

Driving and using machines
You may find that your vision is blurred for a time just after you use Ophtesic. Do not drive or use machines until this has worn off.

3. How to use Ophtesic

Ophtesic will be put on your eye(s) by the doctor before the ophthalmic procedure.

Posology

Your doctor will cover your eye(s) surface and adnexa with the gel 5 minutes before the procedure.
1 gram spread at the surface of the eye is usually enough to get an initial anaesthesia. Additional quantity may be used depending on the size of the eye or the duration of the procedure.

1 gram corresponds roughly to one third of a tube.

The doctor will not exceed one tube per eye or per single procedure.

The gel may be reapplied to maintain anaesthetic effect.

Mode of administration

1. Tear-off the paper cover, remove the tube and the nozzle from the sterile blister and check its integrity.

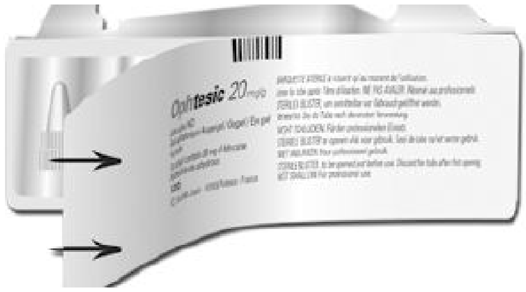

2. Assemble both parts by screwing the nozzle on the tube thread, and twist in clockwise direction to pierce the aluminium sealing membrane.

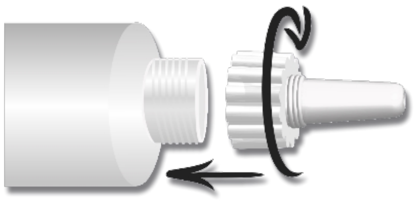

3. Hold the tube pointing down, gently press on the side to release the gel and to cover the eye surface and adnexa.

4. The gel must be kept in place 3 to 5 min before being rinsed. Do not touch the eye or eyelid with the tip of the nozzle.

5. Discard the tube and the blister after use.

4. Possible side effects

Like all medicines, this medicine can cause side effects, although not everybody gets them.

The following side effects have been described with an unknown frequency:
- conjunctival redness
- corneal epithelial changes
- eye burning sensation
- corneal punctate inflamation
- corneal swelling
- headache

Reporting of side effects
If you get any side effects, talk to your doctor. This includes any possible side effects not listed in this leaflet. You can also report side effects directly via the national reporting system listed in Appendix V. By reporting side effects, you can help provide more information on the safety of this medicine.

5. How to store Ophtesic

Keep this medicine out of the sight and reach of children.

Do not use this medicine after the expiry date which is stated on the tube and the box after 'Exp'. The expiry date refers to the last day of that month.
Do not store above 25°C
The product is for single-use and must be discarded immediately after use.

6. Contents of the pack and other information

What Ophtesic contains

- The active substance is lidocaine: 1 gram of gel contains 20 mg of lidocaine hydrochloride (anhydrous).
- The other ingredients are: Hypromellose (E464) type 2910, sodium hydroxide (E524), hydrochloric acid (E507) (for pH adjustment), and water for injections.

What Ophtesic looks like and contents of the pack

Ophtesic is a clear and colour less gel supplied in a 3.5 g tube.

Box of 1, 20, or 100 tubes

Marketing Authorisation Holder

LDD
93, rue Jean Jaurès
92800 Puteaux
France

Manufacturer

RECIPHARM KARLSKOGA AB
Bjökbornsvägen 5 – Box 140
691 33 Karlskoga
Sweden

This leaflet was last approved in MM/YYYY.
