# Supplementary material for: Comparing the in vitro efficacy of chlorhexidine and povidone-iodine in the prevention of post-surgical endophthalmitis
Source: J Ophthalmic Inflamm Infect. 2024 May 23;14:20. doi: 10.1186/s12348-024-00404-2 (PMC11116284; doi:10.1186/s12348-024-00404-2)
Supplement: Supplementary file 5 — Supplementary Material 5 [file 12348_2024_404_MOESM5_ESM.pdf]

FICHA TÉCNICA

1. NOMBRE DEL MEDICAMENTO

Clorxil 5 mg/ml solución cutánea

2. COMPOSICIÓN CUALITATIVA Y CUANTITATIVA

Cada ml de solución contiene: 5 mg de Clorhexidina Digluconato.

Para consultar la lista completa de excipientes ver sección 6.1.

3. FORMA FARMACÉUTICA

Solución cutánea
Solución transparente e incolora

4. DATOS CLÍNICOS

4.1 Indicaciones terapéuticas

Desinfección de heridas, drenaje de heridas, úlceras, ostomías, mucosas y sondaje vesical.

4.2 Posología y forma de administración

Posología

Población pediátrica

Aplicar sobre la zona afectada una o dos veces al día.

Adultos

Aplicar sobre la zona afectada una o dos veces al día.

Forma de administración

Uso cutáneo.
Empléese sin diluir. Limpiar y secar la zona a tratar antes de aplicar el medicamento.
Aplicar directamente sobre la zona afectada o bien sobre una gasa. Dejar actua y secar.

MINISTERIO DE SANIDAD, POLITICA SOCIAL E IGUALDAD
Agencia española de medicamentos y productos sanitarios

4.3 Contraindicaciones

Hipersensibilidad a la clorhexidina o a alguno de los excipientes incluidos en la sección 6.1
No utilizar en ojos, ni oídos.

4.4 Advertencias y precauciones especiales de empleo

- Uso externo sobre la piel.No ingerir.
- En caso de contacto accidental con ojos u oídos, lavar inmediatamente con abundante agua.
- Aunque la absorción de clorhexidina a través de la piel es mínima, no puede excluirse el riesgo de efectos sistémicos. Dichos efectos pueden favorecerse en caso de aplicaciones repetidas, por la utilización del producto sobre grandes superficies, en vendaje oclusivo, sobre piel lesionada y en mucosas.
- No debe usarse para la asepsia de zona de punción o de inyección, ni para la desinfección de material quirúrgico.
- No debe utilizarse en caso de heridas profundas y extensas.
- Las ropas que hayan estado en contacto con este medicamento no se lavarán con lejía ni otros hipocloritos, pues se produciría una coloración parduzca en los tejidos, sino con detergentes domésticos a base de perborato sódico.

Población pediátrica

- El producto sólo debe utilizarse bajo prescripcion facultativa en niños menores de 30 meses.
- El uso de soluciones cutáneas de clorhexidina, de base alcohólica o acuosa, como desinfectante de la piel previo a procesos invasivos, se ha asociado con quemaduras químicas en neonatos. En base a los casos notificados y publicaciones disponibles, el riesgo parece mayor en niños prematuros, especialmente en aquellos nacidos antes de la semana 32 de gestación y durante las primeras 2 semanas de vida.

Se debe retirar cualquier material empapado, gasas o pijamas, antes de comenzar con la intervención. No usar elevadas cantidades y evitar que la solución se acumule en los pliegues de la piel, bajo el paciente o que empape las sábanas u otro material húmedo en contacto directo con el paciente. Cuando se aplique vendaje oclusivo a zonas previamente expuestas a Clorxil 5 mg/ml solución cutánea, se debe tener especial cuidado de que no haya exceso de producto antes de colocar el vendaje.

4.5 Interacción con otros medicamentos y otras formas de interacción

- En general: teniendo en cuenta las posibles interferencias (antagonismo, inactivación, etc) debe evitarse el empleo simultáneo o sucesivo de antisépticos, salvo con otros compuestos catiónicos.
- En particular: no debe usarse en combinación ni después de la aplicación de jabones catiónicos, yodo, sales de metales pesados y ácidos.
  Su actividad antiséptica es parcialmente inhibida por productos orgánicos (suero, etc) y por fosfolípidos.

4.6 Fertilidad, embarazo y lactancia

No hay datos o estos son limitados relativos en humanos, pero los estudios de reproducción en animales no han demostrado riesgo para el feto (categoría B según la FDA).

MINISTERIO DE
SANIDAD, POLITICA
SOCIAL E IGUALDAD
Agencia española de
medicamentos y
productos sanitarios

Se desconoce si se excreta en la leche materna, pero no se han descrito problemas en humanos.
Sin embargo, deberá tenerse en cuenta el posible riesgo de efectos sistémicos.

4.7 Efectos sobre la capacidad para conducir y utilizar máquinas

La influencia de Clorxil sobre la capacidad para conducir y utilizar máquinas, es nula o insignificante.

4.8 Reacciones adversas

- Riesgo de efectos sistémicos (ver punto 4.4)
- Reacciones de hipersensibilidad y fotosensibilidad.
- Lesiones traumáticas, intoxicaciones y complicaciones de procedimientos terapéuticos. Quemaduras químicas en neonatos (frecuencia desconocida).

Notificación de sospechas de reacciones adversas

Es importante notificar sospechas de reacciones adversas al medicamento tras su autorización. Ello permite una supervisión continuada de la relación beneficio/riesgo del medicamento. Se invita a los profesionales sanitarios a notificar las sospechas de reacciones adversas a través del Sistema Español de Farmacovigilancia de Medicamentos de Uso Humano: https://www.notificaram.es. .

4.9 Sobredosis

En caso de ingestión accidental, proceder al lavado gástrico y protección de la mucosa gástrica.
Se han descrito casos de hemólisis tras la ingestión de clorhexidina. En caso de hemólisis, puede ser necesaria transfusión sanguínea.

5. PROPIEDADES FARMACOLÓGICAS

5.1 Propiedades farmacodinámicas

Grupo farmacoterapéutico: Antisépticos y desinfectantes. Biguanidas y amidinas: Clorhexidina. Código ATC: D08AC02.

La clorhexidina es un compuesto catiónico biguanídico, utilizado como antiséptico tópico y activo frente a un amplio espectro de microorganismos Gram positivos y Gram negativos. Es más efectivo frente a Gram positivos, que Gram negativos. La clorhexidina, en general no puede considerarse activa frente a bacterias ácido-alcohol-resistentes, hongos, esporas y virus.
La clorhexidina reacciona con los grupos aniónicos de la superficie bacteriana, alterando su permeabilidad.

5.2 Propiedades farmacocinéticas

MINISTERIO DE
SANIDAD, POLITICA
SOCIAL E IGUALDAD
Agencia española de
medicamentos y
productos sanitarios

La absorción de la clorhexidina a través de la piel es mínima. En caso de que se produzca una absorción sistémica, la eliminación se lleva a cabo a través de la bilis o a nivel renal, sin que medie ningún metabolito previo.

La absorción digestiva es prácticamente nula (alrededor del 99% de la dosis ingerida se excreta sin alteraciones en las heces).

La actividad antibacteriana de la clorhexidina en la piel persiste durante varias horas después de su aplicación.

5.3 Datos preclínicos sobre seguridad

Los estudios de toxicidad realizados en animales de experimentación con clorhexidina, demostraron que su toxicidad es prácticamente nula en las condiciones propuestas. Los estudios de carcinogénesis, mutagénesis y teratogénesis no han evidenciado signos de dicha actividad.

6 . DATOS FARMACÉUTICOS

6.1 Lista de excipientes

Agua purificada

6.2 Incompatibilidades

Este medicamento es incompatible con los derivados aniónicos (jabones, etc) ya que la clorhexidina se comporta como un catiónico; por ello, dicho principio activo precipita a pH superior a 8 en presencia de numerosos aniones.

6.3 Periodo de validez

2 años.

Desechar a los 3 meses de la apertura del envase.

6.4 Precauciones especiales de conservación

No requiere condiciones especiales de conservación.

6.5 Naturaleza y contenido del envase

Clorxil 5mg/ml solución cutánea se presenta en:

Frascos blancos de HDPE con tapón obturador de HDPE/LDPE, en las siguientes presentaciones:

<u>Envases unitarios:</u>

MINISTERIO DE SANIDAD, POLITICA SOCIAL E IGUALDAD
Agencia española de medicamentos y productos sanitarios

1 Frasco de100ml
1 Frasco de 250ml

Envases clínicos:

100 Frascos de 10ml
50 Frascos de 50ml
50 Frascos de 100ml
50 Frascos de 250ml
20 Frascos de 500ml
Puede que solamente estén comercializados algunos tamaños de envases.

6.6 Precauciones especiales de eliminación y otras manipulaciones

La eliminación del medicamento no utilizado y de todos los materiales que hayan estado en contacto con él, se realizará de acuerdo con la normativa local.

7. TITULAR DE LA AUTORIZACIÓN DE COMERCIALIZACIÓN

LABORATORIOS BOHM, S.A.
C/ Molinaseca 23-25. Polígono Industrial Cobo Calleja.
28947 Fuenlabrada (Madrid)
España
91 642 18 18

8. NÚMERO(S) DE AUTORIZACIÓN DE COMERCIALIZACIÓN

9. FECHA DE LA PRIMERA AUTORIZACIÓN/ RENOVACIÓN DE LA AUTORIZACIÓN

Septiembre 2022

10. FECHA DE LA REVISIÓN DEL TEXTO

Septiembre 2022

La información detallada y actualizada de este medicamento está disponible en la página web de la Agencia Española de Medicamentos y Productos Sanitarios (AEMPS) http://www.aemps.gob.es.

MINISTERIO DE
SANIDAD, POLITICA
SOCIAL E IGUALDAD
Agencia española de
medicamentos y
productos sanitarios
